# The Impact of Different Factors on the Quality and Volatile Organic Compounds Profile in “Bryndza” Cheese

**DOI:** 10.3390/foods9091195

**Published:** 2020-08-29

**Authors:** Jana Štefániková, Viera Ducková, Michal Miškeje, Miroslava Kačániová, Margita Čanigová

**Affiliations:** 1 AgroBioTech Research Centre, Slovak University of Agriculture in Nitra, Tr. A. Hlinku 2, 949 76 Nitra, Slovakia; michal.miskeje@uniag.sk; 2 Department of Technology and Quality of Animal Products, Faculty of Biotechnology and Food Sciences, Slovak University of Agriculture in Nitra, Tr. A. Hlinku 2, 949 76 Nitra, Slovakia; viera.duckova@uniag.sk (V.D.); margita.canigova@uniag.sk (M.Č.); 3 Department of Fruit Science, Viticulture and Enology, Faculty of Horticulture and Landscape Engineering, Slovak University of Agriculture in Nitra, Tr. A. Hlinku 2, 949 76 Nitra, Slovakia; miroslava.kacaniova@gmail.com; 4 Department of Bioenergy, Food Technology and Microbiology, Institute of Food Technology and Nutrition, University of Rzeszow, Cwiklinkiej 1, 35601 Rzeszow, Poland

**Keywords:** “bryndza” cheese, electronic nose, gas chromatography, volatile organic compounds, microbiota

## Abstract

The aim of this study was to evaluate the influence of different factors on the basic physicochemical and microbiological parameters, as well as volatile organic compounds of traditionally (farm) and industrially produced “bryndza” cheese. The samples were obtained from eight producers in different areas of Slovakia during the ewe’s milk production season, from May to September. The physicochemical parameters set by the legislation were monitored by reference methods. The “bryndza” cheese microbiota was determined by using the plate cultivation method. There was analysis of volatile organic compounds carried out by electronic nose, as well as gas chromatography mass spectrometry. Seasonality and production technology (traditional and industrial ones) are the main factors that affect the standard quality of “bryndza" cheese. Lactic acid bacteria were dominated from bacterial microbiota, mostly presumptive lactococci, followed presumptive lactobacilli and enterococci. The numbers of coliform bacteria were higher in traditionally produced “bryndza” cheese than in industrially produced “bryndza” cheese. The presence of *Dipodascus geotrichum* was detected in all samples. There were key volatile organic compounds such as ethyl acetate, isoamyl acetate, 2-butanone, hexanoic acid, D-limonene, and 2,3-butanedione. The statistically significant differences were found among “bryndza” cheese samples and these differences were connected with the type of milk and dairies.

## 1. Introduction

The traditional Slovak ewe’s milk product is “bryndza” cheese or local cheese “oštiepok” [1,2]. Slovak “bryndza" cheese is natural, white, spreadable cheese, and manufactured by the traditional method. It is recognized in the European Union by Protected Geographic Indication (PGI) status as cheese produced in specified mountainous regions of Slovakia [3], where unpasteurized ewe’s milk is processed immediately after milking by renneting at 29–31 °C for 30 min, using chymosin or chymosin–identical rennet [4], and the cheese grain is formed into the lump cheese. The lump cheese is drained at 18–22 °C for 24 h and is left to ripen for 3 days at 18–22 °C. The production process continues by further ripening at 12–15 °C for 7–10 days. Ripened ewe’s lump cheese is processed by removing the crust, pressed to remove whey and milled with salt solution (4–6% *w/w*), in order to obtain the specific creamy texture of “bryndza”. The mountainous regions of Slovakia differ in altitude, climate, geological, and vegetation profile and there are some scientific evidences about variability of “bryndza” cheese. There are several studies related to the variability of “bryndza” that are lack of common characteristics of this Slovak cheese [5]. Ewe’s cheese represents a matrix with a specific composition which reflected ewe’s milk, as well as different autochthonous non-starter lactic acid bacteria (NSLAB) that produce typical aroma profile of ewe’s lump cheese, barrelled ewe’s cheese and “bryndza” cheese [4,5]. Kacaniova et al. [6] identified 870 isolates from coliforms, enterococci, lactic acid bacteria (LAB) and yeasts in Slovak “bryndza” cheese by MALDI–TOF MS profiling. The most frequently identified species of gram-negative bacteria were *Hafnia alvei* and *Klebsiella oxytoca.* The most frequently identified species of gram-positive bacteria were *Lactococcus lactis* and *Lactobacillus paracasei*. LAB group was represented by *Lactobacillus*, *Lactococcus* and *Pediococcus*. Pangallo et al. [7] confirmed that microorganisms that belong to the species *Galactomyces candidus* and *Yarrowia lipolitica* as typical yeasts of “bryndza” cheese.

Numerous volatile organic compounds (VOCs) of cheese, including raw milk-based ewe’s cheese are formed by proteolysis and by the subsequent transformation of amino acids [8] to α-keto acids [9]. There has been two different major pathways of amino acid degradation identified in *L. lactis* [10] so far. The first pathway is initiated by an elimination reaction of methionine catalyzed by amino acid lyases, and leading to major sulfur aroma compounds [11,12]. The second pathway is initiated by a transamination reaction catalyzed by aminotransferases and resulting especially in volatile amino acids, branched chain amino acids and methionine [13,14]. The resulting α-ketoacids are then degraded to aldehydes, alcohols, carboxylic acids, esters, methanethiol and other sulfur compounds. The most of these compounds are produced by enzymatic degradation, but some of them are the result of chemical degradation in oxidation [15,16]. The characteristics of cheese (flavor, texture, and color) are influenced by the free fatty acids (FFAs) of the milk, the main products of enzymatic hydrolysis of triacylglycerides by esterases and lipases [17]. The goat and ewe’s milk contain high levels of short-and medium-chain fatty acids, in comparison with cow’s milk. Specifically, short-chain fatty acids are very important because of their low perception thresholds. Some studies point out that milk fatty acid content could be a distinguishing clue between breeds [18]. Partial lipolysis occurs in “bryndza” cheese during its ripening. Additionally, FFAs may participate in catabolic reactions and cause an increase in the amount of aroma compounds such as methyl ketones, esters, alkanes, lactones, aldehydes, and secondary alcohols [19].

VOCs are usually analyzed by gas chromatography (GC) after the extraction or pre-concentration of the volatile fraction. The most exhaustive methods for this purpose are high vacuum distillation (HVT), solvent-assisted aroma evaporation (SAFE) or solid phase microextraction (SPME) [20,21] combined with headspace. Sádecká et al. [20] used SPME with gas chromatography-olfactometry (GC–O) for the determination of volatile odorants in May “bryndza” cheese. There were 25 olfactometric responses from the groups of alcohols, aldehydes, esters, ketones, fatty acids, and hydrocarbons recorded, depending on the degree of cheese maturation and from a GC-O point of view. There was an electronic nose (e-nose) based on a gas chromatography used for aroma profile determination of soft, steamed cheese called “parenica” from Slovakia in a previous study [22].

The aim of this study was to obtain microbiological parameters and parallel information of principal VOCs in “bryndza” cheese produced by 8 Slovak producers with the use of e-nose and a GC-MS. The other goal was to prove a significant impact of the dairies and the type of milk on the content of VOCs in “bryndza” cheese.

## 2. Materials and Methods 

### 2.1. Bryndza Cheese Samples

The samples of “bryndza” cheese were obtained from 8 different producers in Slovakia (B1–8) and detailed characteristics were got from the packages (Table 1). Bryndza cheese samples B1, B2, B4, B6, and B7 were produced in the farm dairies and the samples B3, B5, and B8 were produced in the industrial dairies. All samples were collected monthly from May 2019 to September 2019. Each sample (500 g) was transported to the laboratory at a temperature of 6 °C. There were physicochemical parameters, microbiological quality and volatile organic compounds within one day after the delivery determined in the representative samples. A total of 40 samples were analyzed.

### 2.2. Determination of Physicochemical Properties

Fat was determined according to ISO 3433:2008 [23], dry matter according to ISO 5534:2004 [24]. Fat in dry matter was determined by calculation. pH was determined by pH meter Orion Star A211 (Thermo scientific, Renfrew, UK). The analysis of all physicochemical parameters were replicated three times.

### 2.3. Microbiological Analysis

Ten grams of each sample from each cheese group were put into a sterile bag under aseptic conditions and homogenized in 90 mL of sterile peptone/saline solution (0.87%) for 2 min in a Stomacher bagmixer blender (Interscience, Saint-Nom-la-Bretèche, France). Decimal dilutions were prepared according to ISO 6887–5 (2010) [25].

Coliform bacteria (CB) were determined according to ISO 4832:2006 [26]. Yeasts and moulds (Y) were determined according to ISO 21527-1:2010 [27]. Presumptive lactobacilli (PLb) were determined by enumeration of colonies after anaerobic cultivation on de Man, Rogosa and Sharpe (MRS) agar (HiMedia, Maharashtra, India) for 72 h at the temperature of 37 °C. Presumptive lactococci (PL) were determined by enumeration of colonies after aerobic cultivation on M17 agar (HiMedia) for 72 h at the temperature of 30 °C. Enterococci (E) were determined by enumeration of colonies after aerobic cultivation on Bile Esculine Azide (BEA) agar (Biokar, Allonne, France) for 24 h at the temperature of 37 °C according to ISO 27205:2010 [28]. Microbial counts were performed in triplicate.

### 2.4. Analysis of Volatile Organic Compounds by Electronic Nose

The previously described electronic nose (e-nose) (Heracles II, Alpha M.O.S., Toulouse, France) method [22] was used for volatile organic compounds analysis. There was 2.5 g of sample incubated statically in a 20 mL vial in a thermostat block at the temperature of 50 °C for 15 min (Autosampler, Alpha M.O.S.) and 5 mL volume of the headspace gaseous compounds was withdrawn using a headspace autosampler syringe and dispensed into the e-nose injector for each analysis. The identification of the compounds was performed by matching the measured peaks with *Kovats* retention indices with NIST library (The National Institute of Standards and Technology library) (>50%) by software Alpha Soft V14 (Alpha M.O.S.). Each sample was weighed and placed in three different vials, each one was analyzed once.

### 2.5. Analysis of Volatile Organic Compounds by Gas Chromatography Mass Spectrometry

The head-space solid-phase microextraction (HS–SPME) method previously described [20] was used for sample extraction in a modified version. The amount of 2.5 g of sample was incubated statically in a 20 mL vial in a thermostat block at the temperature of 50 °C for 30 min (CombiPal automated sample injector 120, CTC Analytics AG, Zwingen, Switzerland), with an SPME fibre (1 cm; 50/30 µm DVB/CAR/PDMS) (Supelco, Bellefonte, PA, USA) placed in the CombiPal for each analysis. The fibre was initially conditioned by heating in the SPME Fiber Cleaning and Conditioning Station (placed in the CombiPal) at the temperature of 270 °C for 1 h. SPME extracts were desorbed in the GC injector at the temperature of 250 °C for 1 min and the fibre was cleaned in SPME Fiber Cleaning and Conditioning Station at 230 °C for 10 min.

The relative content (expressed in percentage) of samples was determined by gas chromatography mass spectrometry (GC 7890B–MS 5977A) (Agilent Technologies Inc., Santa Clara, CA, USA) equipped with column DB–WAXms (30 m × 0.32 mm × 0.25 µm; Agilent Technologies Inc., Santa Clara, CA, USA) operating with a temperature program and MS conditions [20]. The identification of compounds was carried out by comparing mass spectra (over 80% match) with a commercial database NIST® 2017, and Wiley library, retention times of reference standards (ethyl acetate, hexanoic acid and isoamyl alcohol) comparison of data on occurrence in cheese from Slovakia with literature [5,20,29]. The relative content of determined compounds was calculated by dividing individual peak area by the total area of all peaks. Peaks under 1% were not counted. Each sample was measured in triplicate.

### 2.6. Statistical Analysis

Compounds identified by e-nose with a discriminant D > 0.900 were selected as significant sensors, based on which the PCA (Principal Component Analysis) was made by Alpha Soft V14 (Alpha M.O.S.) software.

The STATGRAPHICS Centurion (© StatPoint Technologies, Inc., Warrenton, VA, USA) and GraphPad Prism 6.01 (GraphPad Software Incorporated, San Diego, CA, USA) were used for statistical physicochemical, microbiological and GC–MS analysis. The ANOVA method complemented by the Test of Tukey´s Multiple Comparison Test and unpair t-test with a value of *p* < 0.05 was applied.

## 3. Results

### 3.1. Physicochemical Properties of “Bryndza” Cheese

The results of the physicochemical parameters of “bryndza” cheese are shown in Table 2. The content of dry matter and fat in the dry matter are the parameters of bryndza cheese prescribed by the legislation [30]. The variability of “bryndza” cheese dry matter content ranged from 2.34% to 5.19% for farm dairies (B1, B2, B4, B6, B7) samples and from 2.37% to 5.52% for industrial dairies (B3, B5, B8) ones. Higher coefficients of variation were found in the parameter of fat in dry matter, which ranged from 1.75% to 7.98% in “bryndza” cheese from farm dairies and from 2.71% to 9.77% in samples from industrial dairies. Mixed “bryndza” cheese (B3, B8) samples had the lowest average fat content. The pH value of bryndza cheese as an indicator of ripening was more stable in “bryndza” cheese from industrial dairies (coefficient of variation ranged from 0.99% to 1.69%) than in “bryndza” cheese from farm dairies (coefficient of variation ranged from 1.22% to 3.82%).

### 3.2. Microbiological Quality

The counts of microorganisms in the analyzed “bryndza” cheese samples are in Table 3. The coliform bacteria in the “bryndza” cheese produced from pasteurized milk (producer B8) were < 1 log CFU/g. In contrast, the counts of coliform bacteria ranged from 3.46 to 6.78 log CFU/g in “bryndza” cheese produced from unpasteurized ewe’s milk.

Enterococci in the “bryndza” cheese from pasteurized milk reached an average value of 2.37 log CFU/g in the monitored period. Higher counts of enterococci were found in “bryndza” cheese from unpasteurized milk in the case of coliform bacteria than in “bryndza” cheese from pasteurized milk. The counts of enterococci in these samples ranged from 4.59 log CFU/g (“bryndza” cheese sample B5 was made from pasteurized cow’s milk and unpasteurized ewe’s milk) to 7.36 log CFU/g.

Presumptive lactococci were dominated microbiota of “bryndza” cheese. The counts ≥ 7.17 log CFU/g were found in all samples. The counts of presumptive lactobacilli in bryndza cheese from pasteurized milk reached levels 6.56 log CFU/g and from unpasteurized milk ≥ 7.73 log CFU/g.

Yeast counts varied from 4.65 log CFU/g to 6.62 log CFU/g. The differences between the counts of yeasts in “bryndza” cheese from unpasteurized and pasteurized milk are not as significant as in the case of other groups of monitored microorganisms. Species *Dipodascus geotrichum* (before *Geotrichum candidum*) was dominated yeast.

### 3.3. Analysis of VOC

The VOC of different chemical natures and varied sensory descriptors were separated by e-nose, based on head-space gas chromatography with a flame-ionization detector. The twenty VOCs were identified as sensors (markers) with D > 0.900 (Table 4). The aroma profiles of “bryndza” cheese were dominated by esters (7), aldehydes (4), alcohols (4), free fatty acids (2), ketones (2), and one monoterpene.

**Figure 1:**
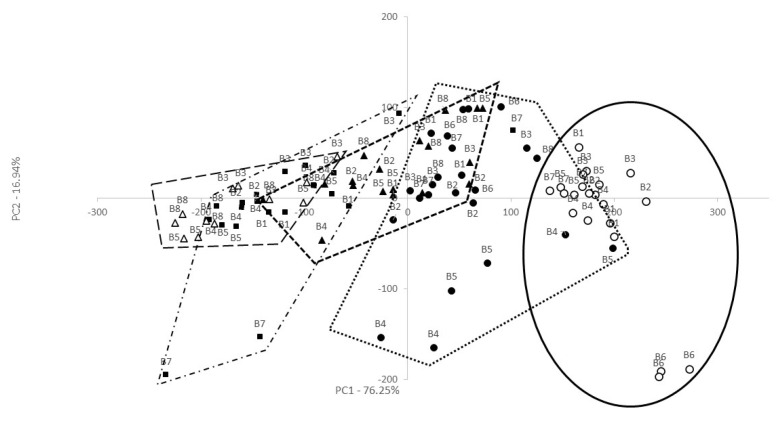
Projection of “bryndza” cheese from 8 producers (B1–8) onto the space defined by the first two principal components (PC1/PC2) based on the e-nose results. Sample groups according to the month: ○—May, ●—June, ▲—July, ■—August, ∆—September.

The dataset of e-nose was in the two-dimensional (2D) PCA plot. The first two principal components accounted for over 90% of total variance indicating that the first two PCs are sufficient enough to explain the total variance of the dataset. When the samples overlap or close to each other, it means they have a similar aroma as it is shown in Figure 1, 40 “bryndza” pieces of cheese from 8 producers divided into 5 groups according to the month of production in PC1 (76.25%). The samples from the May group were far away from the September group, which means that they have significantly different aromas and simultaneously, May and September groups of samples are surrounded by June, July, and August. In general, the PCA result suggested that the e-nose can properly characterize the samples of “bryndza” cheese.

Based on the qualitative analysis of HS–SPME/GC–MS results, a total of 24 VOCs were identified from “bryndza” cheese produced by 8 producers including alcohols (7), free fatty acids (6), esters (4), aldehydes and ketones (4), one monoterpene and one oxime in this study. Data in Table 5 shown the average chemical composition of samples produced during 2019 (from May to September). The compounds with the highest relative contents in “bryndza” were isoamyl alcohol (2.86–10.6%), acetoin (6.16–20.7%), acetic acid (7.42–11.3%), butanoic acid (ND–5.88%), and methoxy-phenyl-oxime (3.74–8.54%).

The six compounds identified by e-nose with a D > 0.900 with ethyl acetate, isoamyl acetate, 2-butanone, hexanoic acid, D-limonene, and 2,3-butanedione were confirmed by GC-MS in this study. Statistical analysis of variance was used to differentiate cheese by evaluation scores such as type of milk, and dairies (farm or industrial) (Table 5). Particularly type of milk had a significant influence on the amounts of identified VOC. The relative chemical composition (TIC% Area) of 2,3-butanediol, 1-methoxy 2-propanol, 2-butanol, acetoin, 2-butanone, acetic acid, octanoic acid, *n*-decanoic acid, ethyl acetate, isoamyl acetate, and phenyl-methoxy-oxime were not significantly influenced by type of milk or dairies. Statistical analysis by the Test of Tukey’s confirmed that TIC% Area of ten compounds was influenced by type of milk. The TIC% Area of β-phenyl ethanol, 2,3-butanedione, 2-phenetyl acetate, and D-limonene was significantly influenced by dairies (*p* ˂ 0.05).

## 4. Discussion

The quality of “bryndza” cheese is affected by many factors, such as the composition of used ewe’s and cow’s milk, the seasonality of ewe’s milk, or the microbiota of used starter culture [31,32,33]. “Bryndza” cheese must have a dry matter content at least 44 wt.% and fat in dry matter of ewe’s “bryndza” cheese should be at least 48 wt.% and in mixed “bryndza” cheese at least 38 wt.% [30].

The reason for fat, fat in dry matter and dry matter content fluctuation in “bryndza” cheese from farm dairies is that the fat content in milk is not standardized in the traditional production. It is known that the fat and dry matter content of ewe’s milk changes naturally during the season [34]. Industrial dairies also use cow’s milk to “bryndza” cheese production, which explains lower fat content in the “bryndza” cheese from these producers (B3 and B8). Most “bryndza” cheese samples from the industrial producer (B3) had lower dry matter content than it is in legislative requirements. The changes in the composition of ewe’s milk and in the produced lump cheese, as well during the season were also reflected in the sensory properties of “bryndza” cheese.

Coliform bacteria are considered as indicators of faecal contamination or poor hygienic conditions in obtaining and processing milk into cheese or low counts of competing LAB. The counts of LAB were high in the analyzed “bryndza” samples, therefore, the coliform bacteria counts indicate poor hygiene in milk processing into cheese. The exception is the counts of coliform bacteria in the “bryndza” cheese from farmers B1 and B2. The differences in the counts of coliform bacteria between “bryndza” cheese made from unpasteurized and pasteurized milk are in line with the results of other studies in this area [35]. Coliform bacteria in “bryndza” cheese have also been found by other authors [7,32,33].

Higher counts of enterococci were found in “bryndza” cheese samples made from unpasteurized ewe’s milk on farms. Some authors [36] even found higher counts of enterococci (8.0 log CFU/g) in “bryndza” cheese made from raw ewe’s milk in comparison with our results. In contrast, there are some results with very similar counts of enterococci (5.11–5.85 log CFU/g) in “bryndza” cheese made from raw and pasteurized milk in published studies [33]. The presence of enterococci in food may not always indicate faecal contamination, but rather, a violation of hygiene and sanitation principles. In the case of some food (cheese and fermented meat products), enterococci are added into them in a targeted way to improve organoleptic properties [37]. Enterococci and yeast in ewe’s cheese made from raw milk contribute to the formation of acetic acid esters [38].

Various genera and species of LAB are present in the microbiota of raw ewe’s milk. Their counts gradually increase during the production of lump cheese and “bryndza” cheese [7]. Starter culture, which is not so varied in genus and species, must be added into the milk after pasteurization to achieve lactic acidification. “Bryndza” cheese made from pasteurized milk may have lower counts of lactic acid bacteria, especially lactobacilli, as it is evidenced by our results and the works of other authors [32]. LAB break down lactose into lactic acid and various aroma compounds such as diacetyl, acetoin, acetaldehyde, or acetic acid [39]. However, they contribute very little to the lipolysis of milk fat. LAB cause the degradation of casein fractions into small peptides and free amino acids by the production of proteinases and peptidases. Amino acids can change into various alcohols, aldehydes, acids, esters, and sulfur compounds, which contribute to the specific aroma of the cheese [40].

Yeast is a natural microbiota of “bryndza” cheese and acts in the secondary stage of this cheese ripening. It is not surprising that the differences in yeast counts in “bryndza” cheese, made from raw and pasteurized milk, are minimal. *Dipodascus geotrichum* has a dominant position among yeasts in bryndza cheese. This type of yeast grows on the surface of the lump and breaks down lactates, milk fat (free fatty acids are released) and proteins (peptides and amino acids) [41,42]. Some strains of *Dipodascus geotrichum* are able to produce esters and various sulfur compounds, which contribute to the formation of a typical aroma and overall quality parameters of cheese [41].

LAB, enterococci, and yeasts *Galactomyces candidus* [6,7,32,43] play a key role in aroma development during cheese ripening. The VOCs are generated by the enzymatic degradation of amino acids in cheese, especially in cheese containing only LAB. The amino acid transamination is catalyzed by lactococci aminotransferases and this is the first step in the degradation of volatile and branched-chain amino acids, which are precursors of volatile organic compounds [44,45]. The resulting α-ketoacids are then degraded to aldehydes, alcohols, carboxylic acids, esters, methanethiol, and other sulfur compounds [46]. The seven compounds as 2-methyl propanol, ethyl acetate, ethyl butanoate, 2-butanone, isoamyl acetate, hexanoic acid, and butane-2,3-dione were identified in this study by e-nose and gas chromatography-olfactometry, as previously described in “bryndza” cheese from Slovakia [4,5,20,32]. Several of the identified compounds (ethyl acetate, 2-phenyl ethyl acetate, ethyl propanoate, 2-methyl-propanol, 2-phenyl ethanol, 2,3-butanediol, 3-hydroxy-2-butanone (acetoin), 3-methyl butanol, 2,3-butanedione, acetic, butanoic, pentanoic, and octanoic acids) are known to be components of different foreign ewe’s cheese as the Oscypek, Canestrato Pugliese, Fiore Sardo, Torta del Casar, Terrincho, Roncal, Manchego, Pecorino Romano [47,48,49]. Other identified compounds, benzaldehyde [13], 2,7-dimethyl-4,5-octanediol [50] were previously described as secondary metabolites by LAB. Passerini et al. [51] confirmed that strains of *L. lactis* with the citP gene and the citM-G cluster produced a larger amount of VOCs than the strains without this genetic information. Likewise, the quality differences in milk and dairy products from different grazing areas have been previously reported [52,53].

The terpenes composition (limonene, myrcene, carvone) of milk and cheese are directly transferred from ingested botanical species and free fatty acids (acetic, butanoic, pentanoic, octanoic, decanoic, and dodecanoic acids) can also be effective to trace animal management and feeding systems [54]. Fatty acids were the most abundant VOCs in the barrelled ewe’s cheese (intermediate product in the production of winter bryndza) from Slovakia [4] and in the raw ewes’ milk cheese Torta del Casar [47,48] or Feta cheese [52]. The free fatty acids are also precursors of methyl ketones, alcohols, lactones, and esters, so they may play an important role in the global aroma development of cheese [48]. The identified 2-methyl propanal was previously described [55] as milk aromas. The other compounds, *n*-butanol and methoxy-phenyl-oxime were previously identified in cheese produced from pasteurization milk fermented with cultures mixed (*L. bulgaricus* and *Streptococcus thermophilus*) and with *Dregea sinensis* Hemsl. protease [56].

The e-nose technology in this study can detect the fingerprint of VOCs present in the headspace of the “bryndza” sample and “parenica” cheese previously described [22] by means of a gas chromatography principle. There are several studies in which an e-nose method containing 10 metal-oxide semiconductors for characterization of the volatile profile of French cheese types [57], Danish blue cheese [58], or Pecorino cheese were used [59]. These mentioned works used e-nose with sensors and it could not determine and identify concrete VOCs and therefore there was a need to confirm the results by GC methods.

## 5. Conclusions

The composition, microbiological quality, as well as the production of volatile organic compounds, changed during the ewe’s milk season and “bryndza” cheese production by farm and industrial producers from Slovakia. The type of heat treatment of the milk, and the technology of the “bryndza” cheese production, also had an impact on the microbiological quality. There were coliform bacteria, enterococci, presumptive lactococci, presumptive lactobacilli, and yeasts detected. The key VOC were defined in “bryndza” cheese by e-nose and GC–MS. Based on the PCA result, the May samples had significantly different aromas, compared with the September samples.

## Figures and Tables

**Table 1:** Characterization of analyzed bryndza cheese samples.

	B1	B2	B3	B4	B5	B6	B7	B8
Ewe’s Milk	100%	100%	min 50%	100%	min 50% or 100%	100%	100%	min 50%
Raw/Pasteurized Milk	R	R	ewes´ R cows´ P	R	P	R	R	P
Dry Matter	min 44%	UL	min 44%	UL	min 44%, 48%	44%	min 48%	min 44%
Fat in Dry Matter	min 48%	UL	min 48%	UL	min 48%	48%	min 48%	min 48%
NaCl	max 2.5%	max 2.5%	max 2.5%	UL	max 2.5%	max 2%	max 2.5%	1.9%
Producing Area of Slovakia	middle	middle	middle	west	middle	east	middle	middle
Package	plastic foil	plastic foil	plastic foil	plastic foil	paper + aluminium foil	plastic container	plastic foil	plastic foil

UL—unlabeled.

**Table 2:** Physicochemical properties of bryndza cheese (average values, means ± SD).

	B1	B2	B3	B4	B5	B6	B7	B8
F (%)	24.8 ± 2.1	24.9 ± 1.6	19.4 ± 2.5	24.8 ± 1.3	24.3 ± 3.3	26.4 ± 0.4	26.3 ± 3.1	22.4 ± 0.5
DM (%)	47.7 ± 1.1	48.9 ± 1.4	42.5 ± 2.0	54.1 ± 2.8	49.3 ± 2.7	52.0 ± 1.4	53.1 ± 2.2	45.5 ± 1.1
FDM (%)	51.8 ± 3.4	50.9 ± 2.2	45.6 ± 4.5	45.8 ± 1.4	49.1 ± 3.9	50.8 ± 0.9	49.5 ± 4.0	49.4 ± 1.3
pH	5.24 ± 0.2	5.09 ± 0.1	5.34 ± 0.1	5.24 ± 0.1	5.14 ± 0.1	5.21 ± 0.1	5.26 ± 0.2	5.24 ± 0.1

F—fat, DM—dry matter, FDM—fat in dry matter.

**Table 3:** Microbiological quality of bryndza cheeses (values of geomean) (log CFU/g).

	B1	B2	B3	B4	B5	B6	B7	B8
CB	3.77	3.46	6.01	6.78	3.72	6.22	5.27	<1
E	5.35	7.36	7.35	7.34	4.59	7.19	6.47	2.37
PL	8.36	9.15	8.98	9.03	8.98	8.52	8.59	7.17
PLb	7.73	8.98	8.69	8.76	8.48	8.35	8.32	6.56
Y	6.62	6.21	6.26	4.65	6.52	5.96	6.19	5.80
DG	5.75	5.20	5.11	4.33	5.93	5.49	6.06	5.24

CB—coliform bacteria, E—enterococci, PL—presumptive lactococci, PLb—presumptive lactobacilli, Y—yeasts, DG—*Dipodascus geotrichum*.

**Table 4:** Volatile organic compounds in bryndza cheese determined by electronic nose (e-nose) with D > 0.900.

Volatile Organic Compounds		*Kovats*´ Retention Index DB-5 Column	*Kovats*´ Retention Index DB-1701 Column	Sensory Descriptor ^1^
**Ketones**	2-butanone	594	690	butter, cheese, chemical, chocolate, ethereal, gaseous
	2,3-butanonedione	589	690	butter, caramelized, creamy, fruity, pineapple, spirit
Aldehyde	propanal	489	579	ethereal, plastic, pungent, solvent
	butanal	578	668	chocolate, green, malty, pungent
	heptanal	901	986	citrus, fatty, fruity, green, smoky
	2-methyl propanal	522	626	brunt, fruity, green, toasted, spicy, malty, pungent
	furfural	836	978	almond, bread, sweet
Esters	ethyl acetate	614	677	acidic, butter, caramelized, fruity, orange, pineapple, pungent, solvent, ethereal, sweet
	butyl acetate	813	879	banana, bitter, ethereal, green, strong, fruity, pear, pineapple, sweaty, sweet
	ethyl butanoate	800	865	acetone, banana, bubblegum, caramelized, fruity, pineapple, strawberry, sweet
	isoamyl acetate	876	945	banana, fresh, fruity, pear, sweet
	ethyl propanoate	710	766	acetone, fruity, solvent
	methyl 2-methyl butanoate	774	840	apple, chewing gum, fruity, solvent, spirit
Alcohols	2-methyl propanol	626	736	alcoholic, bitter, chemical, glue, leek, lecorice, solvent, winey
	2-propanol	500	602	alcoholic, ethereal
	*n*–butanol	664	779	cheese, fermented, fruity, medicinal
	1-hexanol	868	980	dry, floral, fruity, grassy, green, herbaceous, mild woody
Free Fatty Acids	propanoic acid	739	889	acidic, pungent, rancid, soy
	hexanoic acid	996	1186	cheese, fatty, goat, pungent, rancid, sweaty
Monoterpenes	limonene	1049	1073	citrus, fruity, minty, orange, peely

^1^ Sensory descriptor from AroChemBase database, part of software Alpha Soft V14 (Alpha M.O.S.).

**Table 5:** Average chemical composition (TIC% Area) ^1^ of the bryndza cheese from producers B1–8 produced from May to September 2019 obtained by gas chromatography (GC–MS) analysis.

Volatile Organic Compounds (TIC% Area)		B1	B2	B3	B4	B5	B6	B7	B8
Alcohols	isoamyl alcohol ^a,2^	5.20	4.64	4.07	3.22	10.6	4.34	2.86	4.56
	2,7-dimethyl-4,5-octandiol ^a^	1.19	1.39	1.01	1.06	ND ^3^	0.93	0.72	0.94
	2,3-butanediol	1.19	1.60	3.30	3.94	2.55	2.03	1.40	2.56
	β-phenyl ethanol ^a,b^	0.72	0.75	6.73	0.97	2.40	1.52	1.07	6.82
	dl-erythro-1-phenyl-1,2-propanediol ^a^	1.78	0.64	0.71	0.60	ND	ND	ND	ND
	1-methoxy 2-propanol	ND	ND	ND	ND	1.59	ND	ND	ND
	2-butanol	ND	ND	ND	12.36	ND	ND	ND	ND
Aldehyde	Benzaldehyde ^a^	2.04	0.79	0.55	0.48	ND	ND	0.62	ND
Ketones	acetoin	6.16	11.3	11.8	15.2	18.5	20.7	18.9	10.6
	2,3-butanedione ^b^	ND	2.55	3.55	1.95	2.39	1.31	3.15	3.24
	2-butanone	ND	ND	ND	16.47	ND	ND	2.77	ND
Free fatty acids	acetic acid	7.42	10.4	9.87	11.3	9.42	7.97	8.95	8.74
	butanoic acid ^a^	4.33	4.02	2.00	1.80	2.45	3.01	5.88	ND
	pentanoic acid ^a^	1.15	1.50	1.00	ND	0.74	0.74	1.37	2.69
	hexanoic acid ^a^	3.71	4.04	3.74	2.85	3.96	3.94	6.98	3.66
	octanoic acid	ND	1.76	1.97	1.37	4.18	3.55	4.23	1.52
	*n*-decanoic acid	ND	ND	ND	ND	2.51	ND	2.37	ND
Esters	ethyl acetate	3.10	5.26	6.10	3.63	3.63	8.07	ND	4.65
	2-phenetyl acetate ^a,b^	0.93	1.37	8.07	0.85	ND	0.60	ND	3.34
	acetoin acetate ^a^	ND	0.77	1.97	ND	1.14	0.90	ND	1.05
	isoamyl acetate	ND	ND	1.76	ND	ND	ND	ND	3.04
Monoterpenes	D-limonene ^a,b^	1.05	0.76	0.50	ND	ND	ND	0.71	ND
Oxime	phenyl-methoxy-oxime	8.54	5.69	4.40	4.33	4.20	3.74	4.46	5.15

^1^ Listed are the compounds that represented min. 1% in at least one bryndza cheese. ^2^ Letters in superscript indicates statistically significant difference (*p* ˂ 0.05): ^a^—among samples depending on type of milk; ^b^—among samples depending on dairies. ^3^ ND—not detected.
